# Synthetic RNA–protein modules integrated with native translation mechanisms to control gene expression in malaria parasites

**DOI:** 10.1038/ncomms10727

**Published:** 2016-03-01

**Authors:** Suresh M. Ganesan, Alejandra Falla, Stephen J. Goldfless, Armiyaw S. Nasamu, Jacquin C. Niles

**Affiliations:** 1 Department of Biological Engineering, Massachusetts Institute of Technology, Cambridge, 02139 Massachusetts, USA

## Abstract

Synthetic posttranscriptional regulation of gene expression is important for understanding fundamental biology and programming new cellular processes in synthetic biology. Previous strategies for regulating translation in eukaryotes have focused on disrupting individual steps in translation, including initiation and mRNA cleavage. In emphasizing modularity and cross-organism functionality, these systems are designed to operate orthogonally to native control mechanisms. Here we introduce a broadly applicable strategy for robustly controlling protein translation by integrating synthetic translational control via a small-molecule-regulated RNA–protein module with native mechanisms that simultaneously regulate multiple facets of cellular RNA fate. We demonstrate that this strategy reduces ‘leakiness' to improve overall expression dynamic range, and can be implemented without sacrificing modularity and cross-organism functionality. We illustrate this in *Saccharomyces cerevisae* and the non-model human malarial parasite, *Plasmodium falciparum*. Given the limited functional genetics toolkit available for *P. falciparum*, we establish the utility of this strategy for defining essential genes.

## Abstract (web summary)

Current strategies for regulatory control of gene expression are orthogonal to the host organism mechanisms. Here the authors demonstrate an RNA aptamer controlled system integrated into native regulatory pathways in the parasite *Plasmodium falciparum*.

## Graphical abstract

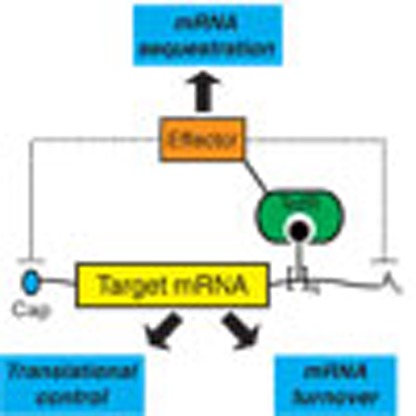

The ability to precisely, reversibly and temporally regulate gene expression is essential for gaining insights into how biological systems function as well as designing biological circuits that direct novel cellular behaviours in synthetic biology applications. In model eukaryotic systems, this can be flexibly achieved at the transcriptional, translational and posttranslational levels. When available, transcription-based regulatory systems are favoured, as they provide access to large dynamic ranges that allow gene expression to be titrated to a level appropriate for the specific application^1^,^2^,^3^. Increasingly, translational and posttranslational control systems are being used to diversify the gene regulation toolkit and fine-tune expression. However, compared with transcription-based systems, the dynamic ranges attainable with these systems are relatively modest, and higher basal expression levels are observed in the repressed state^4^,^5^,^6^,^7^,^8^. This limits their utility as robust and general solutions for controlling gene expression. Thus, straightforward strategies that extend the dynamic range of posttranscriptional control systems would significantly increase the flexibility with which these systems can be broadly used as effective tools.

Towards this goal, we have focused on enhancing the regulatory dynamic range attainable during synthetic control of translation. Current designs emphasize achieving function independent of host cell regulatory mechanisms. Thus, small-molecule-regulated aptamers and riboswitches that directly block translation initiation, and natural or engineered ribozymes that induce *cis*-cleavage of target transcripts have dominated system design^4^,^6^,^7^,^8^. These approaches enable modular systems that function independently of host cell mechanisms, and, in principle, are broadly applicable in different organisms and cell types. While conceptually simple, this framework is restrictive, as it limits opportunities to achieve improved functionality through direct integration of synthetic parts with the diverse mechanisms cells have evolved to robustly regulate translation.

Natively, translation is controlled at multiple levels. Initiation via cap-dependent recruitment of factors critical for ribosome assembly, messenger RNA (mRNA) subcellular sequestration and turnover are key and highly regulated steps. Many proteins that play key roles in regulating these processes have been previously characterized^9^,^10^. For example, eukaryotic translation requires recognition of the mRNA cap structure by eIF4E, which recruits the eIF4F complex and leads ultimately to ribosome assembly and translation. However, the efficiency of this process is modulated by eIF4E-binding proteins, which block eIF4F recruitment and inhibit translation. Similarly, decapping and deadenylation enzymes, such as Dcp1p and Pop2p, respectively, in *S. cerevisiae*, that control mRNA turnover have been characterized^11^. In many instances, homologues of these characterized factors are found widely across eukaryotes, suggesting they play highly conserved roles. Thus, synthetic gene regulation schemes can potentially be significantly enhanced if effective strategies for integration with native translation control components can be devised. Furthermore, conservation of the core translation machinery and regulatory components across diverse organisms suggest that this should be attainable without sacrificing design modularity and portability between different cell types and organisms.

Herein, we describe a straightforward strategy for directly integrating synthetic and native translation regulatory components to achieve improved inducible control of gene expression. By fusing the Tet repressor protein (TetR) to various translation regulatory effectors, we achieve robust anhydrotetracycline (aTc)-dependent regulation of target transcripts via TetR aptamers genetically encoded in either their 5′- or 3′-untranslated regions (UTRs). The regulatory dynamic ranges achieved come close to that of some transcription-based approaches when these aptamer elements flank the target open reading frame. We also illustrate the generality of our approach across two unrelated organisms, namely, the model yeast *S. cerevisiae* and the human malarial parasite, *P. falciparum*. In the latter organism, where few tools are available for studying gene function, we show that this technology can be used to stringently classify genes essential for parasite survival.

## Results

### Molecular design principles

**Figure 1:**
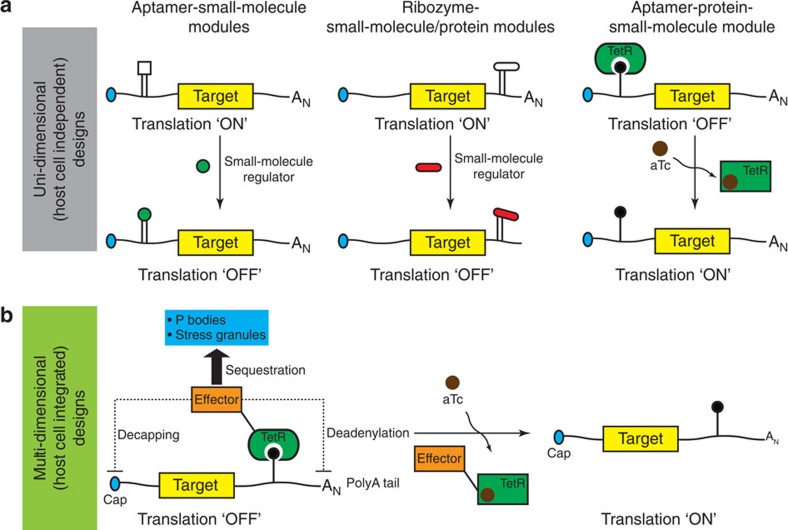
Integrating a synthetic RNA–protein interaction module with native translation control mechanisms. (**a**) Several previously implemented approaches for posttranscriptional regulation of gene expression in eukaryotes are summarized. These are classified as ‘uni-dimensional' since primarily one activity determines the availability or accessibility of a target transcript for translation. (**b**) Schematic of the proposed strategy for integrating a synthetic RNA–protein module with host cell translation control mechanisms to achieve ‘multi-dimensional', posttranscriptional regulation of gene expression. Fusing the regulatory protein in the synthetic module to host cell factors involved in controlling RNA turnover, degradation and sequestration is proposed as an approach for extending the dynamic range and reducing leaky expression of the synthetic system. Direct control of gene expression is achieved by toggling the synthetic RNA–protein interaction via a small-molecule inducer.

Existing strategies for achieving synthetic control of eukaryotic translation rely mostly on aptamer–small molecule interactions^6^, inducible ribozymes^7^,^8^ and RNA–protein interactions^4^,^5^,^12^. These control systems are ‘uni-dimensional' in design in that the required RNA aptamer element is strategically positioned within a target transcript to predominantly direct a single outcome, such as disruption of translation initiation or induced RNA degradation (Fig. 1a). Such designs emphasize modularity and portability, but likely at the expense of achieving maximal robustness, by limiting intimate coupling between the synthetic module and native regulatory mechanisms.

Examination of natural translation control mechanisms, however, reveals that a diverse set of activities simultaneously converge on a regulated transcript to achieve stringent control over its translation^9^,^10^. We hypothesized that synthetic translation control systems that more closely recapitulate nature's ‘multi-dimensional' strategy might inherently be more robust, and achieve significantly improved regulatory outcomes than existing uni-dimensional designs. To establish proof of concept, we reasoned that a broadly useful framework should consist of a foundational RNA–protein interaction module in which: (1) a small molecule directly controls the interaction, and allows for transcription-independent translational control; and (2) the protein component is readily engineered to create fusions that bridge the foundational RNA–protein module with native cellular translation control components (Fig. 1b). Successfully validating this approach would provide immediate access to diverse mechanisms used natively to stringently control the cellular fate of RNA, and establish a new and easily adaptable paradigm for designing multi-dimensional translation control systems with improved functional characteristics.

### TetR fusion proteins conditionally regulate translation

We selected a previously defined aptamer-TetR protein module that is regulated by tetracycline analogues^13^ as the foundational framework for this study. This module has been used in a uni-dimensional context (Fig. 1a, right panel) to control translation in the model organism, *S. cerevisiae*^5^ and the unrelated human malarial pathogen, *P. falciparum*^14^. As such, direct quantitative comparisons between uni- and multi-dimensional implementation can be made to rigorously quantify improvements in functionality achieved via the latter design. Critical to addressing generalizability of this concept, this selection also facilitates assessment of this multi-dimensional regulatory framework to deliver enhanced translational control in an unrelated organism.

In choosing TetR fusion partners, we emphasized two criteria that the selected proteins must satisfy. Namely, these must (1) have been previously implicated in regulating translation initiation, mRNA stability and/or turnover or mRNA sequestration; and (2) have clearly identifiable homologues in other eukaryotes, and, in particular, *P. falciparum*. The latter criterion provides a stringent test of our central principle in a non-model organism where very little basic mechanistic evidence supporting specific posttranscriptional and translational regulatory mechanisms has been directly elucidated.

**Figure 2:**
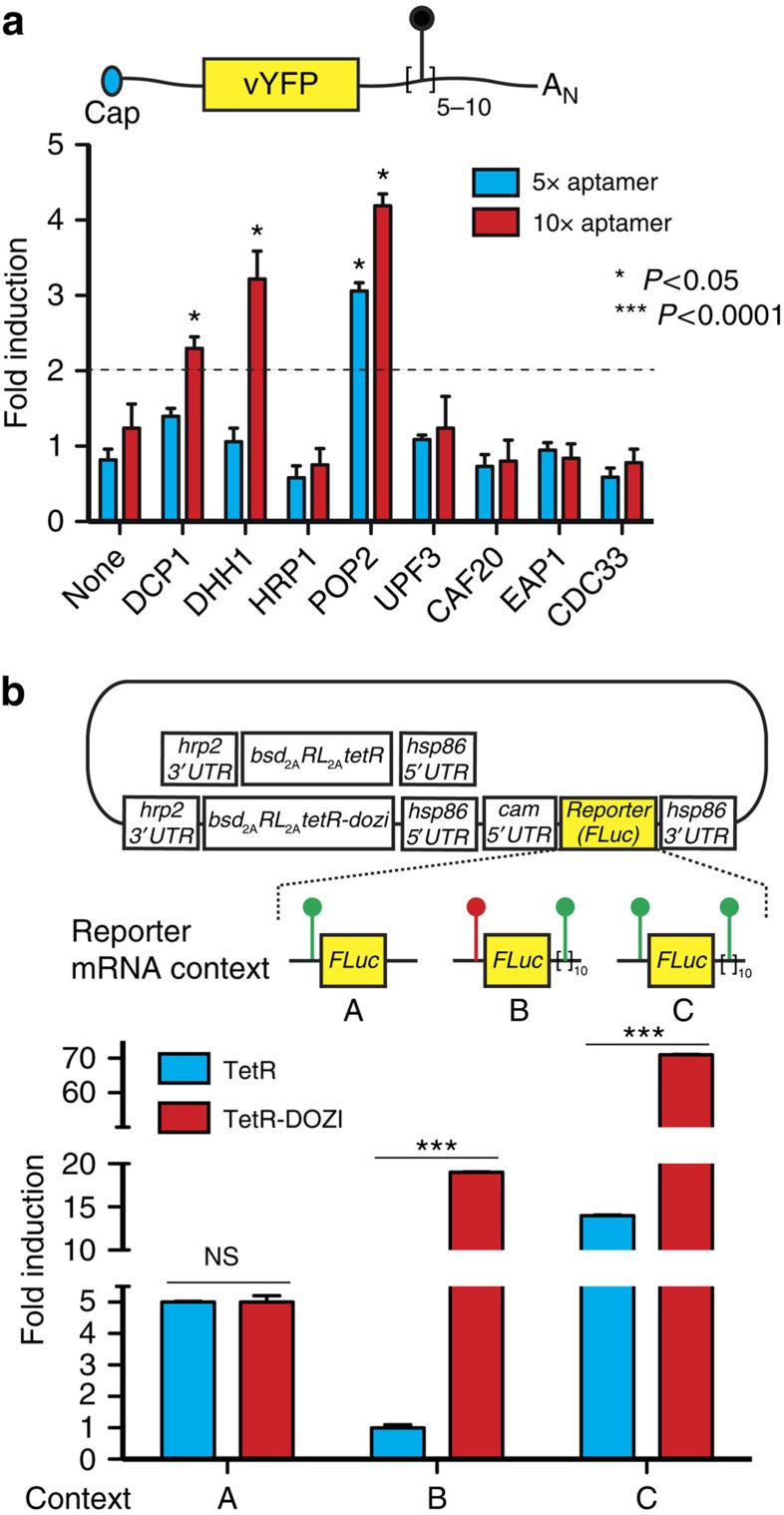
TetR fusion proteins enhance translational regulation in both *S. cerevisiae* and *P. falciparum*. (**a**) In *S. cerevisiae*, various proteins fused to TetR were evaluated for their ability to enhance doxycycline-dependent regulation of a vYFP reporter via either five or ten tandem TetR aptamers positioned within the 3′-UTR. (**b**) In *P. falciparum*, regulated expression of a FLuc reporter by TetR and the TetR-DOZI fusion was tested. Three reporter contexts were examined, namely: (i) single TetR aptamer in the 5′-UTR only; (ii) single mutated TetR aptamer in the 5′-UTR and 10 × TetR aptamers in the 3′-UTR; and (iii) single TetR aptamer in the 5′-UTR and 10 × TetR aptamers in the 3′-UTR. Functional and mutated (no binding to TetR) aptamers are illustrated as green and red lollipops, respectively. In both panels, fold induction is calculated as the ratio of reporter expression in the induced state (+aTc) relative to that in the repressed state (−aTc). Data shown are the mean±s.d. from biological triplicates, and are representative of two (*S. cerevisae*) and three (*P. falciparum*) independent experiments. *P<0.05; ***P<0.0001 by *t*-test. NS=Not significant.

The success of this approach is predicated on identifying fusion proteins that preserve both the inducible interaction between TetR and its aptamer, and the partner's native role in regulating translation. We performed a focused screen in *S. cerevisiae* to identify potential candidates by creating fusions of TetR to Dcp1p, Dhh1p, Hrp1p, Pop2p, Upf3p, Caf20p, Eap1p and Cdc33p, which have all previously been implicated in some aspect of native translational regulation in yeast^11^,^15^,^16^,^17^,^18^. We made a reporter construct in which either five or ten tandem TetR aptamers (referred to hereafter as 5 × and 10 × aptamer arrays) were positioned within the 3′-UTR of a venus yellow fluorescent protein (vYFP)-coding sequence^19^ (Fig. 2a) to identify TetR fusions that mediated doxycycline (Dox)-inducible changes in vYFP expression relative to TetR alone. Notably, TetR by itself displays no inherent inducible behaviour when its cognate aptamers are installed in a 3′-UTR context (Fig. 2a). This is consistent with studies of native translational control mediated by protein binding within the 3′-UTR, where factors that modulate cap-dependent translation initiation, decapping, polyadenylation/deadenylation or mRNA sequestration, as examples, must be recruited to achieve a regulatory outcome^9^,^20^. We did not examine the effects of placing tandem aptamers within the 5′-UTR, since our previous data indicated that increasingly stable secondary structure within this region significantly decreases maximal expression without substantially improving the regulatory dynamic range^5^.

In the 5 × aptamer array reporter context, only TetR-Pop2p resulted in significant Dox-dependent regulation (∼3-fold relative to TetR). In the 10 × aptamer array reporter context, however, TetR-Dcp1p, TetR-Dhh1p and TetR-Pop2p all exhibited Dox-dependent regulation of vYFP expression (∼2–4-fold relative to TetR). All fusions did not yield improved regulation, indicating that simply fusing any protein to TetR is insufficient to achieve an improvement in translational regulation. Overall, these screening data indicate that fusing specific proteins natively involved in regulating translation to TetR can significantly improve the regulatory dynamic range beyond that attainable with TetR alone, while preserving tetracycline analogue-dependent control. These findings provided initial validation that integrating our synthetic RNA–protein module with host regulatory mechanisms could yield improved regulatory outcomes beyond that of the RNA–protein module alone when tested in the identical reporter context.

### Enhanced regulation of gene expression in *P. falciparum*

We focused next on systematically understanding how a selected TetR fusion protein that improved translational regulation outcomes in a model system would function in the non-model eukaryotic pathogen, *P. falciparum*. Our rationale for this approach was twofold. First, native translational regulation in this organism is poorly understood. We reasoned that this choice would provide important insights into the broader applicability of our concept without a need for detailed prior knowledge of fundamental translation control mechanisms in the host organism. Second, the toolkit for robust conditional gene expression in *P. falciparum* is small, with limitations in both dynamic range and basal leakiness^14^,^21^,^22^,^23^ We reasoned, therefore, that a host-integrated synthetic control module with substantially enhanced regulatory dynamic range would also have immediate practical value as a functional genetics tool.

Of the three functional fusions identified during our yeast screens, we selected the TetR-Dhh1p context for detailed evaluation in *P. falciparum*. Several factors influenced this decision. First, we wished to use the *P. falciparum* homologue rather than assuming the yeast protein functions equivalently in *P. falciparum*. BLAST sequence analysis comparing the yeast and *P. falciparum* homologues of Dcp1p, Dhh1p and Pop2p revealed that Dhh1p and PF3D7_0320800 (DOZI) had very high sequence identity and similarity (68% and 80%, respectively), and were similar in size (506 versus 433 amino acids, respectively) (Supplementary Fig. 1a). In contrast, Dcp1p and Pop2p and their *P. falciparum* homologues are much less conserved (Supplementary Fig. 1b,c). The *P. falciparum* homologues are also large proteins with poorly conserved regions that potentially encode functionally important regulatory information. Therefore, creating functional fusions based on these proteins might entail initial screens to identify suitable fragments that preserve both critical biochemical information for efficient integration with the native machinery and TetR function upon fusion. We reasoned that a TetR-DOZI fusion closely reflected the context validated in yeast, and, together with its compact size, would be an ideal primary candidate to test in *P. falciparum*.

Second, the Dhh1p homologue from the related rodent malarial parasite, *P. berghei*, has been implicated in translational regulation. Deleting this protein (referred to as development of zygote inhibited or DOZI based on the observed phenotype) dysregulates the stability of many transcripts that are normally repressed during asexual-stage development in blood and derepressed during sexual-stage development in the mosquito vector^24^. A detailed mechanism of how DOZI functions in this process is lacking, though *in vitro* translation studies using the *P. falciparum* DOZI suggests a role in regulating eIF4E-dependent translation^25^. This is likely only a partial explanation of this protein's function *in situ*, however, since it seems to operate within a larger regulatory protein complex that is still to be functionally defined^26^. Furthermore, the DDX6 helicase protein family, to which Dhh1p and DOZI belong, is implicated in regulating diverse aspects of RNA biology including decapping, degradation and sequestration^11^,^27^,^28^. Last, Dhh1p homologues in several model organisms including *Caenorhabditis elegans*, *Drosophila melanogaster*, *Xenopus laevis* and humans are all very highly conserved (Supplementary Fig. 1a). Therefore, proof of concept with this TetR fusion would support broad applicability beyond our two primary test organisms.

We first assessed whether TetR-DOZI functions to regulate translation in *P. falciparum*, and created several dual expression cassette reporter plasmids (Fig. 2b). One cassette uses a calmodulin promoter (*Pf*CAM) to transcribe a mRNA-encoding firefly luciferase (FLuc) with regulatory TetR aptamers in the 5′-UTR only, 3′-UTR only or both 5′- and 3′-UTRs to test TetR-dependent translational regulation. The second cassette drives expression of TetR or TetR-DOZI, *Renilla* luciferase (RLuc) and the Blasticidin S deaminase (*bsd*) selection marker from a multi-cistronic transcript using the viral T2A ‘skip' sequence^29^. The *RLuc* is used as an internal reference signal in our quantitative luciferase assays. We site-specifically integrated^30^ these reporter plasmids at the *cg6* chromosomal locus in *P. falciparum* NF54^attB^ (ref. 31), and obtained clonal parasites by limiting dilution. We grew parasites either in the absence or presence of aTc, and measured inducible FLuc expression in the various regulatory contexts described.

These data showed that both TetR and TetR-DOZI regulated FLuc expression by ∼5-fold in an aTc-dependent manner when a single aptamer is present within the 5′-UTR (Fig. 2b, context A). Thus, TetR-DOZI functions indistinguishably from TetR in this context. Next, we examined the degree of regulation achieved with TetR or TetR-DOZI when a 10 × aptamer array is placed within the 3′-UTR, and a non-functional mutated aptamer is placed in the 5′-UTR of the FLuc reporter (Fig. 2b, context B). With TetR, we observed no detectable aTc-dependent regulation of FLuc expression (fold regulation ∼1), which is consistent with our observation in the yeast experiments (Fig. 2a). With TetR-DOZI, however, aTc regulated FLuc expression by ∼19-fold (Fig. 2b). Thus, TetR-Dhh1p in yeast and TetR-DOZI in *P. falciparum* both substantially enhance aTc-inducible regulation of gene expression beyond what is attainable with TetR by itself.

Next, we tested whether combining regulation by a single aptamer in the 5′-UTR and a 10 × aptamer array in the 3′-UTR (Fig. 2b, context C) would synergize to increase the overall dynamic regulatory range attainable. With TetR alone, we observed ∼8–14-fold aTc-dependent regulation of FLuc expression. This represented a ∼2–3-fold increase in dynamic range over that observed during TetR regulation mediated via a single aptamer in the 5′UTR. Thus, while TetR did not directly mediate regulation via aptamers in the 3′-UTR alone (Fig. 2b), an increase in fold regulation occurred when 5′- and 3′-aptamers flank the reporter gene. Bridging interactions are believed to bring the 5′- and 3′-UTRs of eukaryotic transcripts into close proximity with each other during translation^32^. This finding could reflect increased TetR recruitment by the 10 × aptamer array to create locally high TetR concentrations in proximity to the 5′-UTR, such that the fractional occupancy of the regulating 5′-aptamer by TetR is higher than when no 3′-UTR array is present and TetR can only be recruited from a relatively dilute cytoplasmic pool. Intriguingly, with TetR-DOZI, aTc-dependent regulation increased substantially to ∼45–70-fold in the dual-aptamer configuration (Fig. 2b). The improved regulatory outcome seen with TetR-DOZI relative to TetR is consistent with the gain of translation regulation via the 3′-UTR aptamer array enabled by the DOZI component of the fusion protein.

### Enhanced regulation is independent of 5′-UTR context

**Figure 3:**
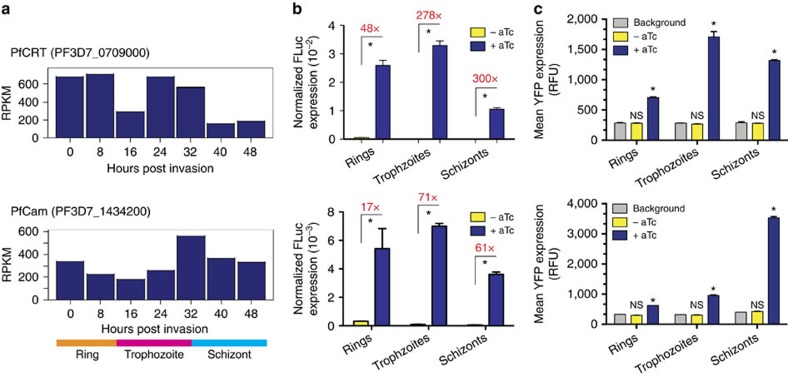
The TetR-DOZI– aptamer module provides robust control of gene expression. (**a**) RNA-Seq data from PlasmoDB showing the stage-dependent transcription profile for two native parasite promoters, *Pf*CRT (PF3D7_0709000) and *Pf*CAM (PF3D7_1434200). (**b**) Stage-dependent FLuc expression normalized to RLuc expression (non-regulated internal reference) in the absence and presence of aTc for both *Pf*CRT (top) and *Pf*CAM (bottom) promoter contexts. The regulatory dynamic range (ratio of aTc induced to basal expression) is shown in red for ring-, trophozoite- and schizont-stage parasites. (**c**) Stage-dependent mean YFP expression in the absence and presence of aTc for both *Pf*CRT (top) and *Pf*CAM (bottom) promoter contexts. Both the FLuc and YFP reporters are controlled by a single TetR aptamer in the 5′-UTR and 10 tandem TetR aptamers in the 3′-UTR. Fold-induction values were not calculated from the fluorescence reporter expression data as the signal to noise is relatively low, and in the repressed state the fluorescence signal is effectively at the autofluorescence background level. Data shown are the mean±s.d. from biological triplicates, and are representative of two to four independent experiments. *P<0.001 by *t*-test. NS=Not significant.

Many genes exhibit highly regulated and presumably functionally important temporal expression profiles during the parasite's 48 h intraerythrocytic developmental cycle (IDC)^33^,^34^. Therefore, we examined whether enhanced regulation is achieved at different time points within the IDC, while using promoters having distinct temporal expression profiles. We selected the *P. falciparum* chloroquine resistance transporter (*Pf*CRT; PF3D7_0709000) and *Pf*CAM (PF3D7_1434200) promoters as representative candidates. These exhibit peak transcriptional activity in early (ring)- to mid-(trophozoite), and late (schizont) stages, respectively (Fig. 3a).

With both promoters, substantially enhanced regulation was observed when TetR aptamers in the 5′- and 3′-UTRs are used together. With the *Pf*CAM promoter, fold expression upon aTc induction ranged from 20- to 70-fold across the IDC. Intriguingly, in the *Pf*CRT promoter context, the observed fold expression was much higher and ranged from ∼50 to 300 fold across the IDC (Fig. 3b). In general, the fold regulation was lowest in early-stage parasites, but regulation by the TetR-DOZI fusion exceeded that by TetR alone^14^. Interestingly, regulation increased more substantially in later-stage parasites. This pattern of regulation is likely not simply due to delayed expression of the regulatory TetR-DOZI fusions, as the *Pf*HSP86 promoter driving expression of this construct is constitutively high across the IDC (http://plasmodb.org/plasmo/showRecord.do?name=GeneRecordClasses.GeneRecordClass&source_id=PF3D7_0708400&project_id=PlasmoDB). We hypothesize that this phenomenon might instead be linked to stage-dependent activation of the native parasite translational regulatory pathway(s) in which the DOZI component of our fusion operates^33^,^34^. Further studies will be required to precisely delineate the DOZI-dependent regulatory pathways in *P. falciparum.* However, it is worth emphasizing that even without a detailed fundamental understanding of these native mechanisms, we consistently achieve substantially enhanced regulation by integrating our synthetic system with this native host translational regulatory mechanism.

We also replaced FLuc with an enhanced YFP (EYFP) reporter gene in both the *Pf*CRT and *Pf*CAM promoter contexts to confirm that other genes could stringently be regulated in this framework. In the repressed state (–aTc), no EYFP signal above background could be detected by flow cytometry for both *Pf*CRT and *Pf*CAM promoter contexts at any point during the IDC. However, aTc induction produced a substantial increase in EYFP signal at all IDC stages (Fig. 3c; Supplementary Fig. 2). In contrast, a single 5′-aptamer within the *Pf*CAM promoter context regulated by TetR alone results in noticeable fluorescence signal above background in the repressed state^14^. Thus, the dual-aptamer configuration with a host-integrated TetR-DOZI regulatory module stringently represses an orthogonal reporter beyond what is attainable with the standalone TetR module. These data indicate that: (1) DOZI-enhanced translational regulation is not restricted to a specific IDC stage; (2) enhanced regulation is achieved independent of the promoter context and expression profile; and (3) adequate concentrations of TetR-DOZI are produced throughout the IDC to achieve robust regulation.

### Leaky expression is reduced with the TetR-DOZI system

A frequent limitation of posttranscriptional regulatory schemes is the challenge of reducing leaky expression, as this can adversely impact the practical utility of these tools for both functional genetics and synthetic biology applications. Given the substantial increase in regulatory dynamic range associated especially with the dual-aptamer configuration, we closely analysed the basis for this outcome.

**Figure 4:**
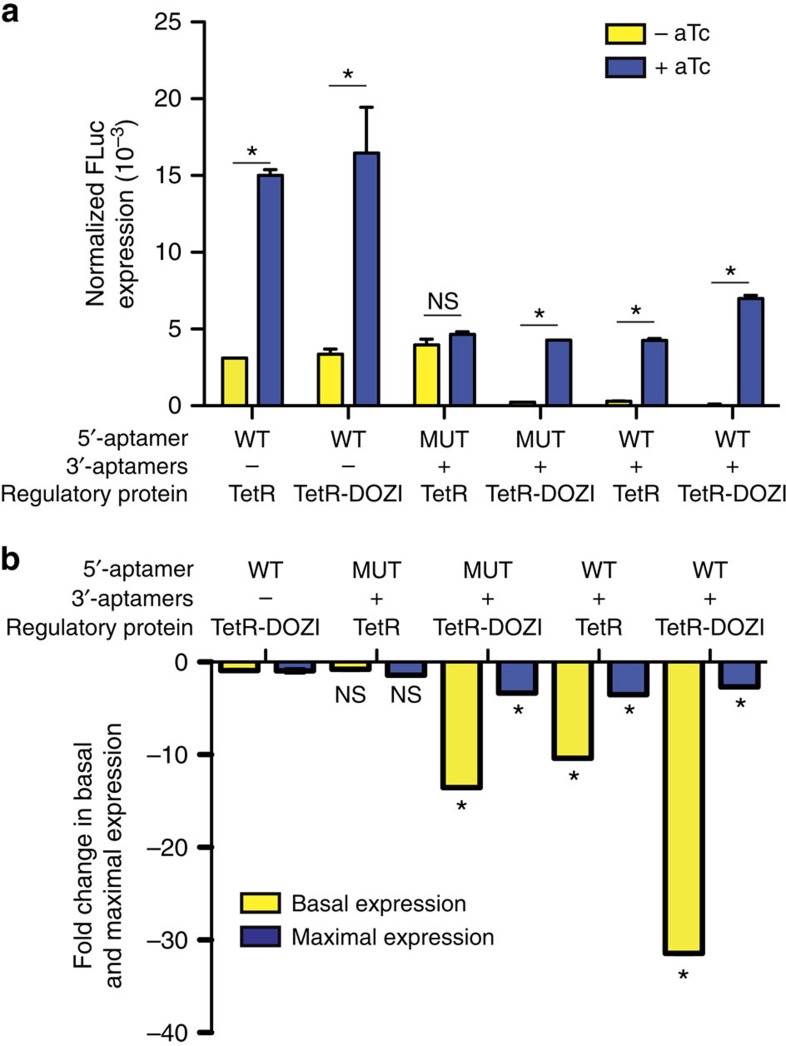
Multi-dimensional synthetic control of translation achieves significant reduction in leaky expression. (**a**) FLuc expression levels in the absence and presence of aTc normalized to RLuc expression (non-regulated internal reference) when TetR and TetR-DOZI are used to regulate FLuc expression via aptamers positioned within the 5′-UTR, 3′-UTR or both as shown. (**b**) Fold change in maximal FLuc expression upon induction (+0.5 μM aTc) and in basal FLuc expression (−aTc) is shown relative to the context in which TetR regulates FLuc expression via a single aptamer in the 5′-UTR. Data shown are the mean±s.d. from biological triplicates, and are representative of two to four independent experiments. *P<0.001 by *t*-test. NS=Not significant.

We observed that inclusion of a 10 × aptamer array within the 3′-UTR of the target transcript reduced maximal reporter expression by ∼2–3-fold (Fig. 4a). This was independent of whether TetR or TetR-DOZI functioned as the regulator, and likely reflects the change introduced into the target transcript. We then compared fold reduction in FLuc expression in the repressed state (−aTc) using TetR with a single 5′-UTR aptamer as a reference (Fig. 4b), and found that substituting TetR for TetR-DOZI in this context had no impact on leaky expression. In the 5′-mutant aptamer and 10 × aptamer in the 3′-UTR context, no reduction in leaky expression is observed with TetR as the regulator. In contrast, leaky expression is reduced 6–12-fold with TetR-DOZI. In the dual-aptamer context, we observed a 7–10-fold reduction in leaky expression with TetR. In contrast, when TetR-DOZI is used as the regulator in this context, we observed a 17–30-fold reduction in leaky expression. Taken together, these data show that integrating the TetR aptamer module with host translational mechanisms mediated through DOZI results in substantially improved regulation of gene expression, and this is predominantly achieved through a reduction in leaky expression. This feature is especially valuable in functional genetics applications where residual protein expression can mask phenotypes mediated by truly essential genes, or in synthetic biology applications where levels of critical components must be stringently regulated to achieve desired circuit performance. In these applications, the reduction in maximal protein expression observed by installing aptamer arrays within the 3′-UTR can be further contextualized. In synthetic designs, the desired protein expression levels can be achieved by choosing appropriate promoters to pair with the aptamer-regulated 3′-UTR. In functional genetics applications, it is conceivable that installing a 3′-aptamer array in the context of a fixed, native promoter may prevent adequate maximal expression of essential genes and preclude recovery of viable organisms for further study. The extent to which this scenario is encountered will only be evident upon broader application of our technology. If encountered, this can be addressed by engineering 5′- and 3′-UTR aptamer-regulated contexts wherein a quantitatively appropriate promoter strength is selected to simultaneously afford functionally adequate maximal gene expression and stringently tunable regulation (Figs 2 and 3). However, our PfATP4 *vide infra* data indicate that this system will be relevant for targeting and studying essential parasite genes via engineering aptamer arrays into the 3′-UTR alone.

Our studies, along with previous data, also suggest a possible model for how regulation might be achieved in this system. We previously showed that a functional aptamer capable of interacting with TetR is absolutely required for aTc-dependent regulation of gene expression, as when a mutated aptamer that does not interact with TetR is used, no aTc-dependent regulation is observed^5^. Alternatively, if expressing the TetR fusion protein alone were sufficient to repress reporter gene expression, this would not be aTc dependent. Furthermore, for the same TetR aptamer configuration, absolute FLuc expression levels in the presence of aTc were indistinguishable in the presence of TetR or TetR-DOZI (Fig. 4a). This indicates that any repression by direct interaction of the DOZI fusion component with the reporter is either minimal or does not have a significant effect on expression. This observation is analogous to our previous study in which TetR fusions to She2p and She3p in yeast were used to conditionally localize target transcripts to daughter cells in yeast^19^. Taken together, these data support a model in which TetR aptamers in a target transcript recruit the TetR-DOZI (or other fusion partner), and this facilitates downstream regulatory outcomes mediated through the fusion partner to occur in a transcript-specific manner. Future studies will be needed to quantitatively dissect which combination(s) of the various translational regulatory roles mediated by DOZI/DDX6 family proteins—such as degradation, deadenylation and sequestration––contribute most to the TetR-DOZI-dependent regulation of targeted transcripts. This information could be useful for designing even more efficient and broadly applicable regulators, in addition to expanding our currently poor fundamental understanding of translational regulation in asexual-stage parasites.

### Another native protein mediates regulation of translation

We next examined whether the extended regulatory dynamic range observed with the TetR-DOZI fusion in *P. falciparum* was limited to DOZI. We selected the *P. falciparum* CAR-1/trailer hitch homologue (CITH) as another fusion partner to test for three key reasons. First, CAR-1 and trailer hitch in *C. elegans* and *D. melanogaster*, respectively, have been implicated as key translational regulators, and this protein family is widely conserved across eukaryotes^35^. Second, like DOZI, CITH has been implicated in regulating translation in *Plasmodium berghei*. Both proteins seem to function within the same translation regulatory complex, though additional orthogonal pathways cannot be excluded^26^. Third, from a practical standpoint, CITH homologues are relatively small proteins, which we reasoned should produce fairly compact and biochemically well-behaved fusions to TetR.

We generated a reporter plasmid in which we replaced TetR-DOZI with a TetR-CITH fusion, and FLuc expression was regulated by aptamers in both 5′- and 3′-UTRs. We then compared aTc-dependent regulation achieved by TetR/5′-aptamer only (benchmark), and TetR, TetR-DOZI and TetR-CITH in the dual-aptamer context. Our data show that TetR-DOZI and TetR-CITH both produced substantially improved translational regulation over TetR alone in either the single or dual-aptamer context (Supplementary Fig. 3). These data show that translational regulators from distinct protein families can be incorporated into our conceptual framework for successfully integrating synthetic and native translational regulation to achieve substantially improved conditional control over target gene expression.

### Establishing gene essentiality in *P. falciparum*

Having established quantitative proof of concept for our proposed strategy, we sought to establish that this approach could have useful applications. To demonstrate this, we used our framework to directly establish for the first time the essential function of a difficult-to-study *P. falciparum* membrane protein, PfATP4 (PF3D7_1211900), which is of increasing interest as a new antimalarial drug target/resistance mechanism. This P-type ATPase protein has been proposed as the putative target of a new, potent and clinically promising antimalarial drug class, the spiroindolones^36^,^37^. However, the available evidence does not definitively demonstrate that loss of PfATP4 function should, in fact, impair parasite survival. Thus, a critical piece of information required for rigorously assigning mechanism of action for a new drug target could not be easily satisfied due to technological limitations. The availability of this information assumes even greater significance given recent data indicating that many diverse chemical scaffolds converge on PfATP4 as a common direct or indirect mechanism of parasite sensitivity and/or resistance to these compounds^38^,^39^.

**Figure 5:**
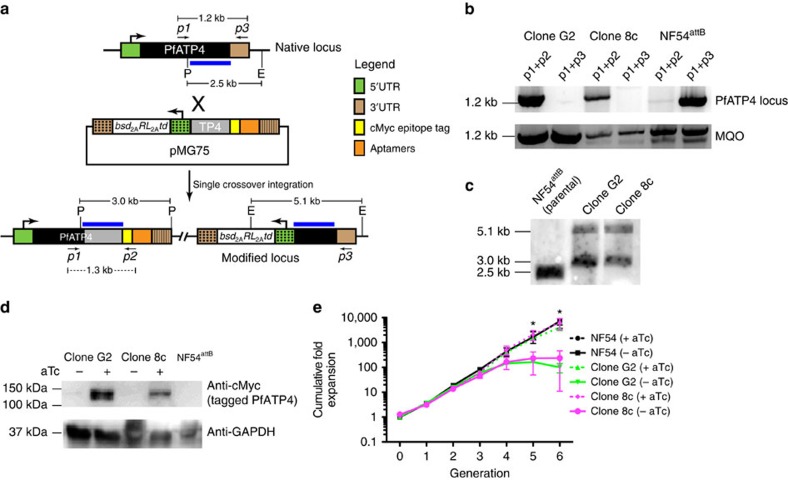
Establishing essentiality of a native parasite gene using TetR-DOZI with a genetically encoded 10 × aptamer array in the 3′-UTR. (**a**) Schematic of the single crossover event to modify the native PfATP4 chromosomal locus to install a C-terminal epitope tag, a 10 × aptamer array and the regulatory TetR-DOZI expression cassette in a single step. Diagnostic PCR primers to assess the status of the locus are designated *p1*–*p3*, and the expected sizes of PCR products derived from the native and modified locus are indicated. The blue bar corresponds to the probe used in Southern blot experiments in which genomic DNA was digested simultaneously with EcoR1 (E) and Pst1 (P). The expected diagnostic band sizes for the native and modified loci are indicated. (**b**) Diagnostic PCR analysis of two isolated clones (G2 and 8c) with modified PfATP4 loci. The malate:quinone oxidoreductase (MQO) gene is used as a positive control for the presence of genomic DNA in all samples tested. (**c**) Southern blot analysis for the parental NF54^attB^ strain and clones G2 and 8c. (**d**) Western blot using anti-cMyc antibody to detect aTc-dependent expression of PfATP4 in clones G2 and 8c. The parental NF54^attB^ strain (no cMyc-tagged PfATP4) is included as a negative control. (**e**) ATc-dependent growth of the parental NF54^attB^ strain and Clones G2 and 8c over multiple generations. Data shown are the mean±s.d. from biological triplicates, and are representative of three independent experiments. *P<0.001 by *t*-test.

To begin addressing this knowledge gap, we implemented our TetR-DOZI system to conditionally regulate PfATP4 expression. Given the large size (∼3.8 kb) of PfATP4, we genetically encoded a 10 × TetR aptamer array in a 3′-UTR context at the native locus as illustrated (Fig. 5a). Clonal parasites were genotyped by PCR (Fig. 5b; Supplementary Fig. 4) and sequencing of the diagnostic products, and Southern analysis (Fig. 5c; Supplementary Fig. 5). We also confirmed stringent aTc-dependent regulation of PfATP4 expression by western blot analysis (Fig. 5d; Supplementary Fig. 6). Altogether, these data confirmed that we had successfully modified the PfATP4 genomic locus and could stringently control the expression of this integral membrane protein target^36^.

We next determined whether downregulating PfATP4 expression adversely impacted parasite growth. We selected two isogenic clones that had been grown continuously in the presence of aTc to ensure maximal PfATP4 expression, and split these into −aTc and +aTc conditions to monitor parasite growth over multiple IDCs. Over the first two IDCs, we observed no significant aTc-dependent difference in growth between the G2 and 8c clones. However, beginning in the third IDC and continuing thereafter, there was a significant decrease in growth of the G2 and 8c clones in the no aTc condition compared with when aTc was present (Fig. 5e). As the parental NF54 line grew similarly both in the absence and presence of aTc, this eliminates an inherent proliferative effect of aTc on parasites as the explanation for the growth phenotype. Altogether, these findings establish, for the first time, that loss of PfATP4 function is indeed deleterious to blood-stage parasite growth.

Here we demonstrate that synthetic RNA–protein modules can be effectively integrated with native cellular mechanisms for controlling translation, and in so doing, more robust regulation of target gene expression can be achieved. While preservation of modularity and cross-organism compatibility criteria has typically motivated designs intended to minimize interactions with native control mechanisms, we show that these properties can be preserved even while intentionally pursuing host-cell-integrated designs. A key outcome of this conceptual framework is a substantial reduction in leaky/basal expression, which has been a major challenge in leveraging robust translation control tools for functional genetics and synthetic biology applications in eukaryotes.

We envision this strategy will be broadly useful as the principles for implementation are straightforward and apply in unrelated organisms, as illustrated here. Many posttranscriptional regulatory proteins operating either through the same or distinct mechanisms have been described^11^. These can serve as potential library components that can be systematically evaluated to establish a core set that can serve as ‘gateways' for efficiently accessing specific modes of native translational regulation. By focusing on components with high protein sequence conservation and presumably regulatory mechanism(s) across different genera, a high rate of successful portability among organisms should be attainable. Novel and orthogonally regulated synthetic RNA–protein interaction pairs can be efficiently discovered using existing methods^4^,^13^. We envision that implementing such orthogonal units using the framework validated here will facilitate multiplexed, stringent and directly tunable control of translation to motivate increasingly sophisticated and robustly controlled functional genetics and synthetic biology applications.

## Methods

### Molecular cloning

Plasmids used in this study were cloned by restriction/ligation, yeast homologous recombination or Gibson assembly methods^29^. All restriction enzymes were purchased from New England Biolabs and reagents were from Sigma-Aldrich, Research Products International, GoldBio Technology or Alfa Aesar, unless otherwise specifically mentioned. PCR amplification of parasite DNA was performed on either crude lysates from the parasite fractions or purified DNA templates using Hemo KlenTaq mixed 15:1 (v:v) with PfuTurbo (Agilent). Supplementary Tables 1 and 2, respectively, summarize the plasmids and primers used in this work. Plasmid DNAs for transfections were prepared using the Xtra Midi Kit (Clontech). Plasmids were propagated in DH5α bacteria, and grown on solid or in liquid media at 30 °C to minimize the potential for spontaneous truncations of the 3′-UTR TetR aptamer arrays.

### Plasmid DNA constructs sequences and sources

GenBank files for the constructs used in this study are provided as supplementary files. *FLuc* is wild-type FLuc DNA carrying the K549E mutation for cytosolic targeting in yeast.

### Yeast inducible expression assays

The various TetR-containing regulatory proteins were cloned as C-terminal fusions to TetR in the YCpSUP yeast expression vector^5^. We created a vYFP reporter plasmid with either 5 and 10 tandem repeats of the TetR aptamer 5–1.2 in the 3′-*UTR* by replacing the non-fluorescent vYFPΔ in vYFPΔ–5–1.2(5 × ) and vYFPΔ–5–1.2(10 × )^19^, respectively, with vYFP. *S. cerevisiae* W303-1B cells harbouring both a repressor and an appropriate reporter plasmid were grown to saturation at 30 °C in Synthetic Defined Media #1 (SD1) (6.7 g l^−1^ yeast nitrogen base without amino acids (RPI), 20 mg l^−1^ adenine, 30 mg^−1^l lysine, 100 mg^−1^l leucine, 20 mg l^−1^ histidine)+20 g l^−1^ glucose. Cells were diluted 1:80 into SD1+20 g l^−1^ raffinose and grown for 4 h. Glucose (to repress TetR or TetR fusion protein expression) or galactose (to induce TetR or TetR fusion expression) was added to 20 g l^−1^, and cells were grown 16 h at 30 °C with shaking before measurement. To measure vYFP expression by flow cytometry, cells were grown in triplicate either in the presence of glucose (±22 μM doxycycline) or galactose (±22 μM doxycycline), and analysed on a C6 Flow Cytometer (Accuri). For all samples, ∼50,000 events were captured and vYFP fluorescence measured in the FL1 channel.

### *P. falciparum* culture and transfection

The *P. falciparum* NF54^attB^ parasites were a gift from David Fidock (Columbia University). These were grown in human erythrocytes (Research Blood Components, Boston, MA) at 5% haematocrit under 5% O_2_ and 5% CO_2_ in RPMI 1640 media supplemented with 5 g l^−1^ Albumax II (Life Technologies), 2 g l^−1^ NaHCO_3_, 25 mM HEPES-K pH 7.4, 1 mM hypoxanthine and 50 mg l^−1^ gentamicin. For transfections, we used 50–60 μg of each plasmid per 200 μl packed red blood cells (RBCs), adjusted to 50% haematocrit. We used a Bio-Rad Gene Pulser II to either directly electroporate DNA into ring-stage parasites^40^ or preload uninfected RBCs^41^. For direct electroporation of ring-stage parasites, 0.2-cm electroporation cuvettes were loaded with 0.3 ml of parasitized RBCs at 50% haematocrit and 50 μg of plasmid DNA in incomplete cytomix solution that contains 120 mM KCl, 0.15 mM CaCl_2_, 10 mM K_2_HPO_4_/KH_2_PO_4_, 25 mM HEPES, 2 mM EGTA and 5 mM MgCl_2_. Electroporation conditions were 0.31 kV and 960 μF. For spontaneous DNA uptake, eight square-wave pulses of 365 V for 1 ms, separated by 100 ms were used. Transfected parasites were selected with 2.5 mg l^−1^ Blasticidin S beginning 4 days after transfection. In the case of *Bxb1*-mediated integration, no selection for the integrase-expressing plasmid was applied. In allelic replacement experiments, drug on/off cycles (28 days each) were completed until the desired integration event was detectable by PCR. Parasites were cloned by limiting dilution thereafter and genotyped by PCR, sequencing of the diagnostic product and Southern analysis.

### Induction experiments and luciferase assays

Ring-stage parasites were tightly synchronized using 0.3 M alanine for three consecutive IDCs before induction. Synchronized parasites were induced using 0.5 μM aTc at ring-stage and allowed to go through a complete IDC before collecting ring-, trophozoite- and schizont-stage samples for luciferase assays. Firefly and RLuc measurements were made with the Dual Luciferase Assay kit (Promega) on a GloMax 20/20 luminometer (Turner Biosystems).

### Western blot

Approximately, 10^6^ late trophozoite-stage parasites were lysed by saponin treatment (0.5 g l^−1^ saponin in PBS). These lysates were prepared for western blot by heating in Laemmli sample buffer at 95 °C for 10 min. After separation by SDS–polyacrylamide gel electrophoresis, proteins were transferred to a polyvinylidene difluoride membrane and probed with a rabbit monoclonal antibody (71D10) against the cMyc tag (Cell Signaling Technology, catalogue no 2278 S; 1:2,000 dilution) or rabbit polyclonal antibody against GAPDH (Abcam, ab9485; 1:1,000 dilution). Blots were imaged using a horseradish peroxidase-coupled secondary antibody and SuperSignal West Femto substrate (Thermo Scientific, catalogue no 34095).

### Flow cytometry

Ring-stage parasites were synchronized and analysed by flow cytometry at the trophozoite stage. Cells were stained for nucleic acid content with 1 μM SYTO 61 (Life Technologies) and analysed using an Accuri C6 flow cytometer (BD Biosciences). EYFP and SYTO 61 signals were monitored in the FL1 and FL4 channels, respectively. Parasites were selectively analysed by gating on events with high FL4 signal intensity. Adding 0.5 μM aTc to either uninfected RBCs or parasites not expressing EYFP had no effect on FL1-H signal intensity.

### Southern blot

Southern blots were carried out using genomic DNA isolated from schizont-stage parasites at ∼2–5% parasitemia in a 30 ml, at 2% haematocrit culture using the QIAamp DNA blood mini kit (Qiagen). RBCs were lysed using a 0.1% saponin solution. Between 2 and 3 μg of genomic DNA was restriction enzyme digested overnight with PstI and EcoRI (New England Biolabs), and probed with a PfATP4 PCR product obtained using primers SMG291 and SMG292. The probe was labelled with biotin-11-dUTPs using the Pierce Biotin Random Prime kit (Thermo Scientific). Blots were processed using the TurboBlotter kit (Whatman) for transfer and the North2South kit (Thermo Scientific) for development.

### Growth assays

Parasites were synchronized to rings using 0.3 M alanine in 10 mM HEPES (pH 7.4) at day 0, adjusted to 1% parasitemia and seeded in 96-well microtiter plates in quadruplicate at 2% haematocrit in 200 μl of RPMI complete media in the presence or the absence of aTc (0.5 μM). The parental NF54^attB^ strain and engineered PfATP4 lines were all included in the assay. Expansion was measured over six IDCs, and samples were analysed every 48 h to determine parasitemias at the end of each IDC. After each measurement, all cultures were split by the same dilution factor required to keep the pre-invasion parasitemia of the control lines below 2% to avoid culture over-expansion. Parasitemias were measured by incubating the cells with 1:1,000 dilution of SYBR Green I for 15 min at 37 °C, before flow cytometry on an Accuri C6 instrument (BD Biosciences) and analysis of data collected in the FL1 channel.

## Additional information

**How to cite this article:** Ganesan, S. M. *et al*. Synthetic RNA–protein modules integrated with native translation mechanisms to control gene expression in malaria parasites. *Nat. Commun.* 7:10727 doi: 10.1038/ncomms10727 (2016).

## Supplementary Material

Supplementary Information Supplementary Figures 1-6 and Supplementary Tables 1-2
