# Supplementary material for: Synthetic RNA–protein modules integrated with native translation mechanisms to control gene expression in malaria parasites
Source: Nat Commun. 2016 Mar 1;7:10727. doi: 10.1038/ncomms10727 (PMC4773503; doi:10.1038/ncomms10727)
Supplement: Supplementary Information — Supplementary Figures 1-6 and Supplementary Tables 1-2 [file ncomms10727-s1.pdf]

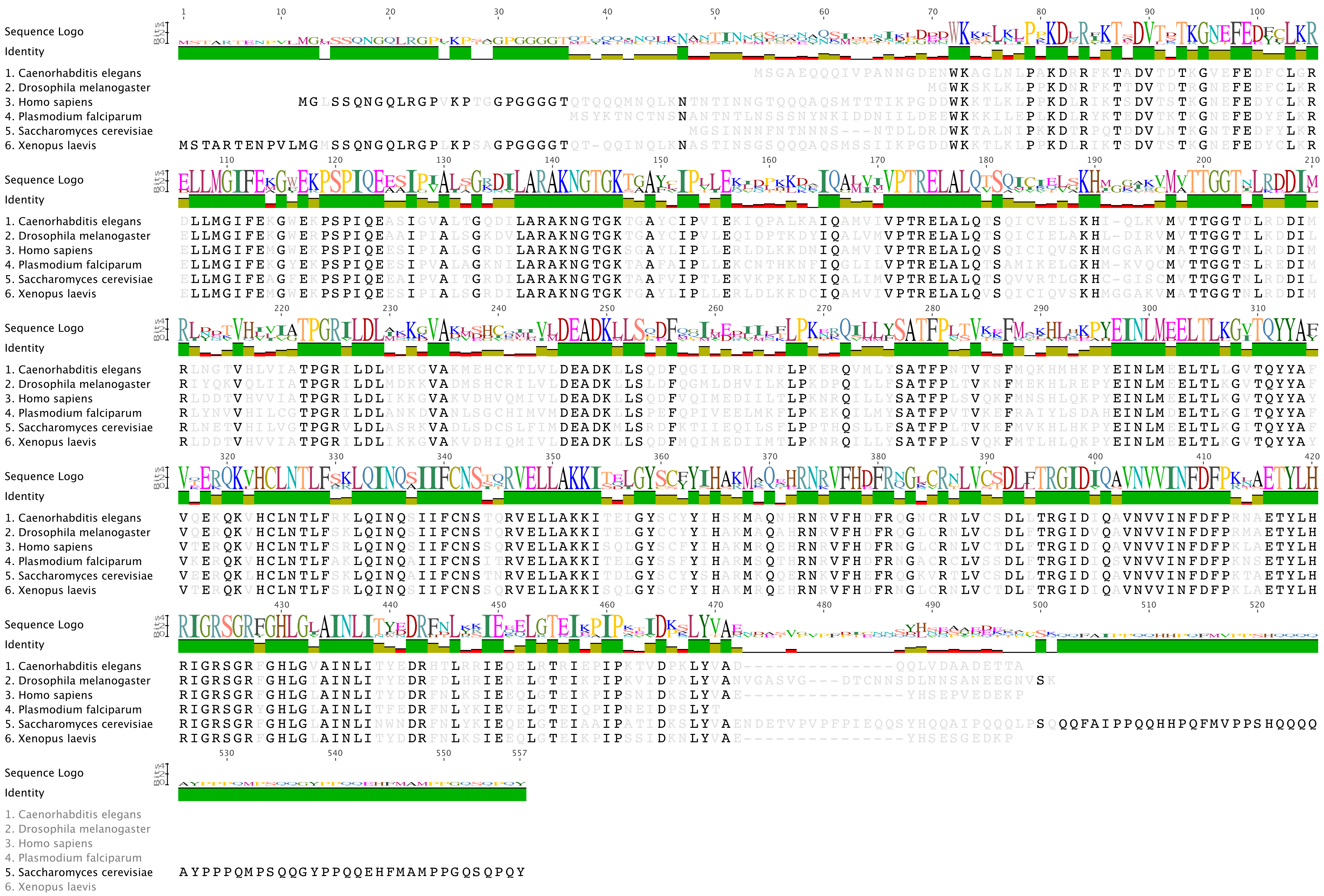

Supplementary Figure 1a. Sequence comparison of several protein homologs from various model and non-model organisms that have been implicated in translational regulation. MUSCLE alignments of the Dhh1p homologs from *S. cerevisiae* with those from *C. elegans*, *D. melanogaster*, *H. sapiens*, *P. falciparum* and *X. laevis*.

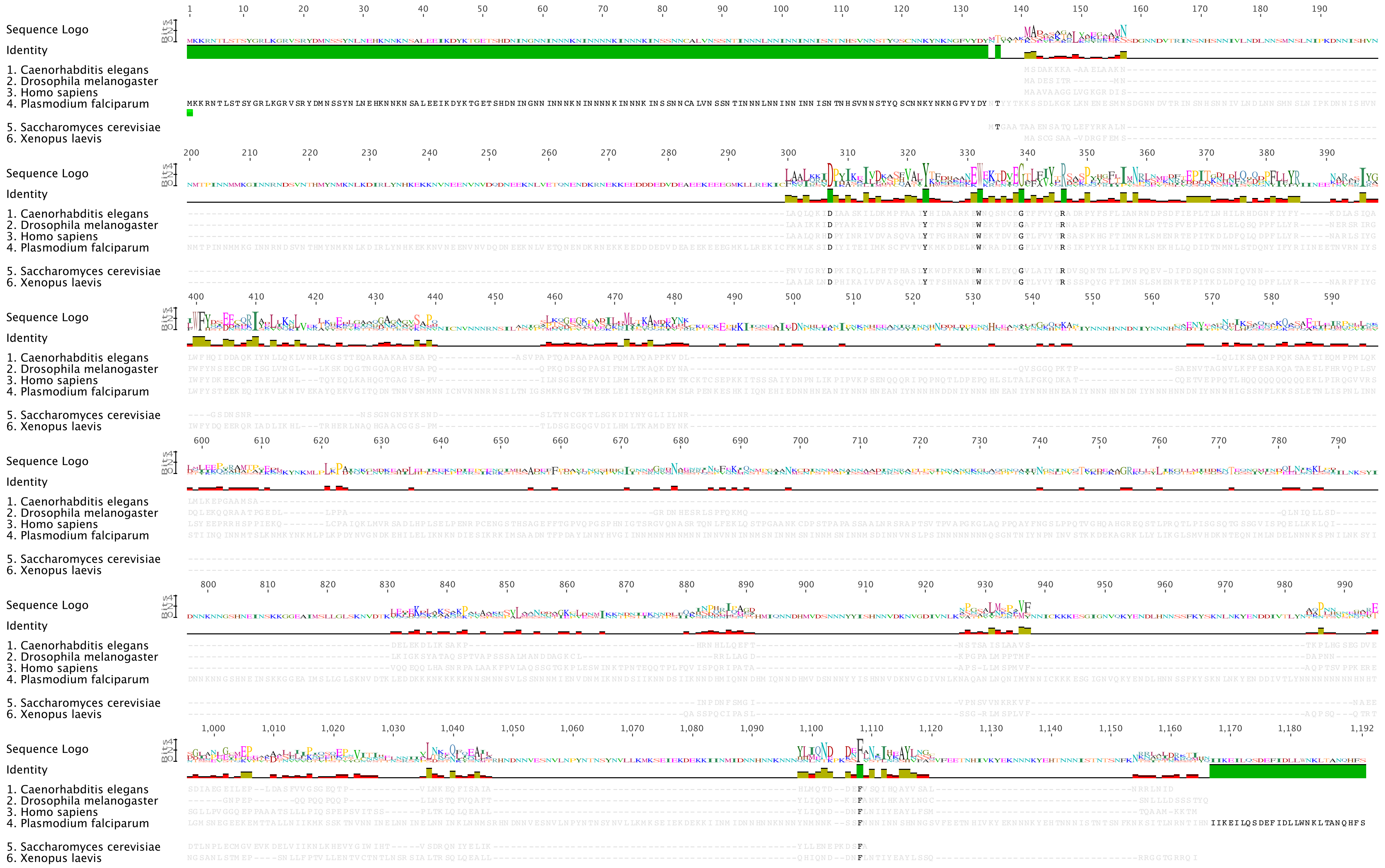

Supplementary Figure 1b. Sequence comparison of several protein homologs from various model and non-model organisms that have been implicated in translational regulation. MUSCLE alignments of the Dcp1p homologs from *S. cerevisiae* with those from *C. elegans*, *D. melanogaster*, *H. sapiens*, *P. falciparum* and *X. laevis*.

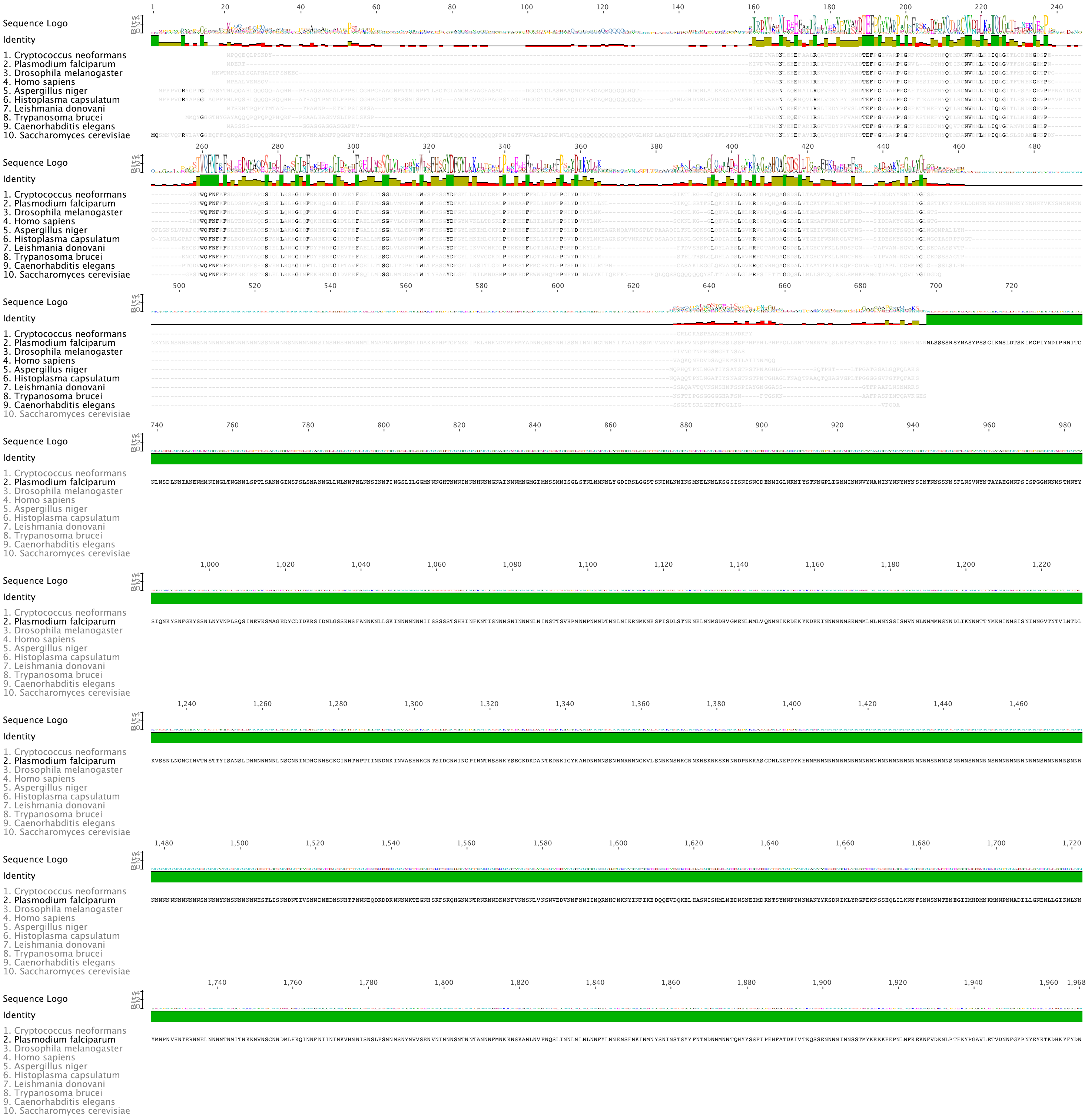

Supplementary Figure 1c. Sequence comparison of several protein homologs from various model and non-model organisms that have been implicated in translational regulation. MUSCLE alignments of the Pop2p homologs from *S. cerevisiae* with those from *C. elegans*, *D. melanogaster*, *H. sapiens*, *P. falciparum* and *X. laevis*.

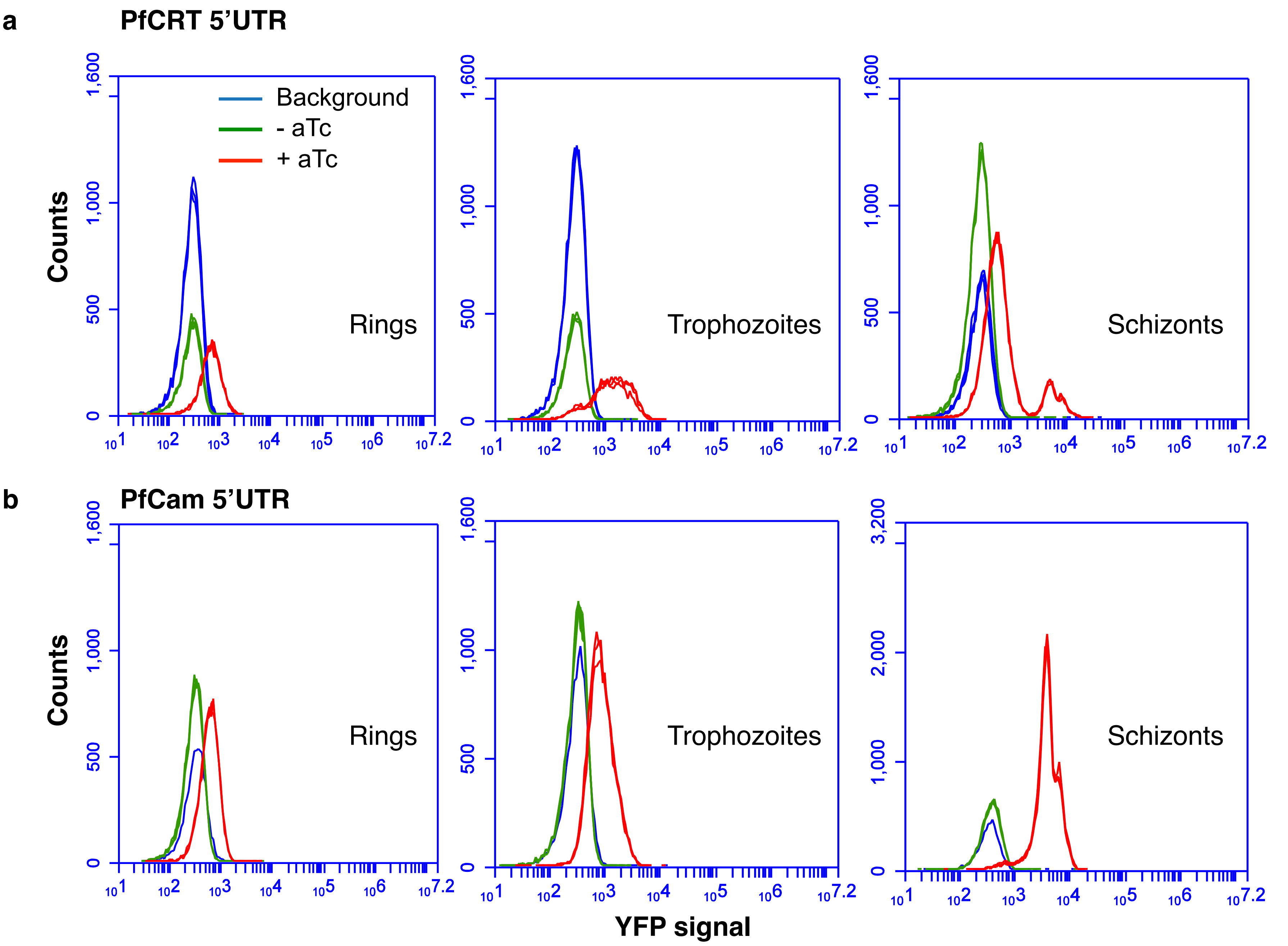

Supplementary Figure 2. Flow cytometry histograms showing parasite developmental stage- and aTc- dependent EYFP expression controlled by TetR-DOZI. (a) PfCRT and (b) PfCAM promoters were used to drive expression of an EYFP reporter gene flanked by a single aptamer within the 5'-UTR and ten tandem aptamers in the 3'-UTR.

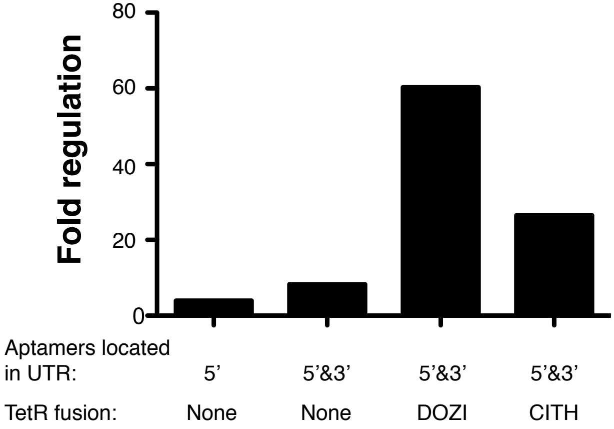

Supplementary Figure 3. TetR fused to the *P. falciparum* CITH homolog improves the regulatory dynamic over TetR alone. TetR-CITH regulation expression is compared with that of TetR and TetR-DOZI using a FLuc reporter flanked by a single aptamer within the 5'-UTR and ten tandem aptamers in the 3'-UTR. Regulation of FLuc by TetR via a single aptamer in the 5'UTR is included for reference.

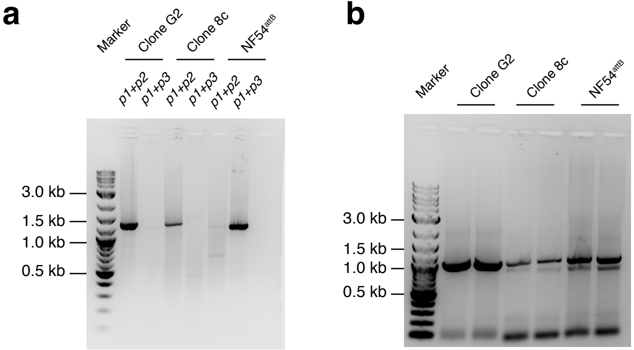

Supplementary Figure 4. **Diagnostic PCR to confirm integration of the TetR aptamer construct at the PfATP4 locus**. (**a**) Two clones (G2 and 8c) and the parental $NF54^{attB}$ strain were analyzed by PCR for integration of the aptamer-containing construct (using the *p1*/*p2* primer pair) or preservation of the original PfATP4 locus (using the *p1*/*p3* primer pair). (**b**) A positive control PCR reaction to amplify a region of the malate-quinone oxidoreductase (MQO) locus was used to demonstrate that all samples tested contained genomic DNA. Marker = 2-Log Ladder (New England Biolabs)

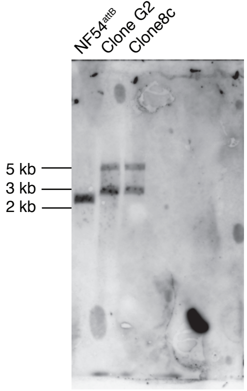

Supplementary Figure 5. **Southern blot to confirm integration of the TetR aptamer construct at the PfATP4 locus**. (**a**) Two clones (G2 and 8c) and the parental NF54^{attB} strain were analyzed to determine whether the aptamer-containing construct had modified the PfATP4 locus. Genomic DNA digested with Pst1 and EcoR1 was probed to reveal products of the expected size for the parental NF54^{attB} line (2.5 kb) and transgenic clones G2 and 8c (5 kb and 3 kb).

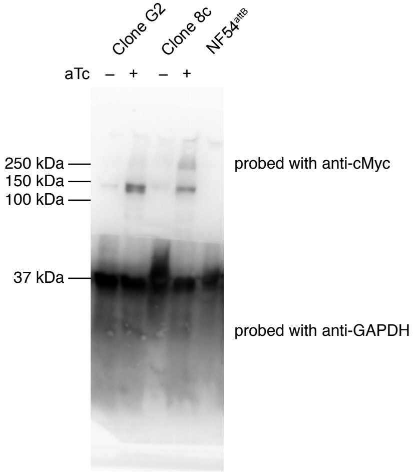

Supplementary Figure 6. **Western blot to assess aTc-dependent regulation of the PfATP4 expression.** Lysates from the transgenic clones G2 and 8c (- and + aTc) and the parental NF54^{attB} strain were analyzed. The top half of the gel was probed with anti-Myc antibody (PfATP4 expression) and the bottom half with anti-GAPDH antibody (loading control).

Supplementary Table 1. List of plasmids used in this study.

Plasmid	Description	GenBank Accession
pMG32	PfCAM promoter driving a FLuc reporter with a single aptamer in the 5'UTR; TetR-DOZI$_{2A}$RLuc$_{2A}$BSD	
pMG53	PfCAM promoter driving a FLuc reporter with a single aptamer in the 5'UTR and 10 tandem aptamers in the 3'UTR; TetR$_{2A}$RLuc$_{2A}$BSD	
pMG54	PfCAM promoter driving a FLuc reporter with a single mutant aptamer in the 5'UTR and 10 tandem aptamers in the 3'UTR; TetR$_{2A}$RLuc$_{2A}$BSD	
pMG56	PfCAM promoter driving a FLuc reporter with a single aptamer in the 5'UTR and 10 tandem aptamers in the 3'UTR; TetR-DOZI$_{2A}$RLuc$_{2A}$BSD	
pMG57	PfCAM promoter driving a FLuc reporter with a single mutated aptamer in the 5'UTR and 10 tandem aptamers in the 3'UTR; TetR-DOZI$_{2A}$RLuc$_{2A}$BSD	
pMG62	PfCRT promoter driving a FLuc reporter with a single aptamer in the 5'UTR and 10 tandem aptamers in the 3'UTR; TetR-DOZI$_{2A}$RLuc$_{2A}$BSD	
pMG83	PfCAM promoter driving a FLuc reporter with a single aptamer in the 5'UTR and 10 tandem aptamers in the 3'UTR; TetR-CITH$_{2A}$RLuc$_{2A}$BSD	
pMG84	PfCRT promoter driving an EYFP reporter with a single aptamer in the 5'UTR and 10 tandem aptamers in the 3'UTR; TetR-DOZI$_{2A}$RLuc$_{2A}$BSD	
pMG92	PfCAM promoter driving an EYFP reporter with a single aptamer in the 5'UTR and 10 tandem aptamers in the 3'UTR; TetR-DOZI$_{2A}$RLuc$_{2A}$BSD	
pMG75	Allelic replacement plasmid to install a C-terminal epitope tag and 10 tandem aptamers in a 3'UTR context at the PfATP4 chromosomal locus to achieve regulated PfATP4 expression by TetR-DOZI.	
pSG372	PfCAM promoter driving a FLuc reporter with a single aptamer in the 5'UTR; TetR$_{2A}$RLuc$_{2A}$BSD	

Supplementary Table 2. List of primers used in this study.

Primer	Sequence	Use
SMG305	tgtggtgatgttgaagaaaatccaggtccatggCCAGTGAGC AAGGGCGAGGAGCTGTTC	Forward primer used to clone EYFP into pMG84
SMG293	gatcttcttcgctaatcagtttctgttcggtaaccTTACTTGTAC AGCTCGTCCATGCCGAGAG	Reverse primer used to clone EYFP into pMG84
SMG307	GTATTAATGGAACAGAAGTAGCTAAAGGA GCATC	Forward primer for detecting integration at the PfATP4 locus
SMG291	ctaaatatatatccaatggcccctttccgggcgcgccttaagCTCA AATATTCAAAAATTTGT ATG TTT CTT GCT TG	Forward primer for cloning the PfATP4 homologous region
SMG292	cacagatcttcttcgctaatcagtttctgttcggtaaccgcATTCTT AATAGTCATATATTTTCTTCTATATATAACC	Reverse primer for cloning the PfATP4 homologous region
SMG286	GTCAATTTTTAGAAGAAAGATATCCTGTAT TTAGA	Forward primer for amplifying the housekeeping MQO gene
SMG358	ATGAATAAGTGGAACATATGGAATAGATA AG	Reverse primer for amplifying the housekeeping MQO gene
SMG357	AAAAACAAGCAAAATTTTTACCACATGTA C	Reverse primer for detecting native PfATP4 locus
SMG342	actaaatatatatccaatggcccctttccgggcgcgccCATTTTG TAAAAAAAATTAAAATATATTTATATAATA TTAT	Forward primer for amplifying PfCAM promoter/5'UTR
SMG343	ccgtccgtatcgacctttctctgcctggattgcttaagTGATATAT TTCTATTAGGTATTTATTATTATAAAATATA AATC	Reverse primer for amplifying PfCAM promoter/5'UTR
SMG255	actaaatatatatccaatggcccctttccgggcgcgccTAGTAGT TGAGTGATTCTATATACATATAC	Forward primer for amplifying the PfCRT promoter
SMG256	ccgtccgtatcgacctttctctgcctggattgcttaagTGTTATAT GTAAGAAATTAAAAAAAACAAAATAATAA ATGAATG	Reverse primer for amplifying the PfCRT promoter
SMG173	ctggtggtggtggtggtggtggtggtggtactagtAGTTATAA AACCAATTGTACGAACT	Forward primer used to amplify

		dozi gene
SMG174	gttaataatgatcctctaccttcaccactaccgctagcGGTATAT AAGGATGGGTCAATTTCG	Reverse primer used to amplify *dozi* gene
SMG164	gtgggtctggtggtggtggtggtggtggtggtggtactagtTCATC TGTGTCAACCTTACCTTA	Forward primer used to amplify *cith* gene
SMG165	catgttaataatgatcctctaccttcaccactaccgctagc ATATGGTGGATATCTATTAAAAGGATTTTG ATTC	Reverse primer used to amplify *cith* gene

Note: Nucleotides shown in lowercase correspond to the homologous region needed for Gibson Assembly cloning, while those in uppercase base pair with the target gene/region.
